# Supplementary material for: Methylation of histone H3K23 blocks DNA damage in pericentric heterochromatin during meiosis
Source: eLife. 2014 Aug 26;3:e02996. doi: 10.7554/eLife.02996 (PMC4141274; doi:10.7554/eLife.02996)

Supplemental MS data for Figure 1D

051010_jrc_TetmicH3_HILIC 5/10/2010 12:20:05 PM

26 ul (~5pmole) Tet mic H3 GluC + 250 fmole AV and ACTH

7-50

051010_jrc_TetmicH3_HILIC #23667-24011 RT: 187.20-189.76 AV: 106 NL: 5.16E6

F: FTMS + c NSI Full ms [300.00-2000.00]

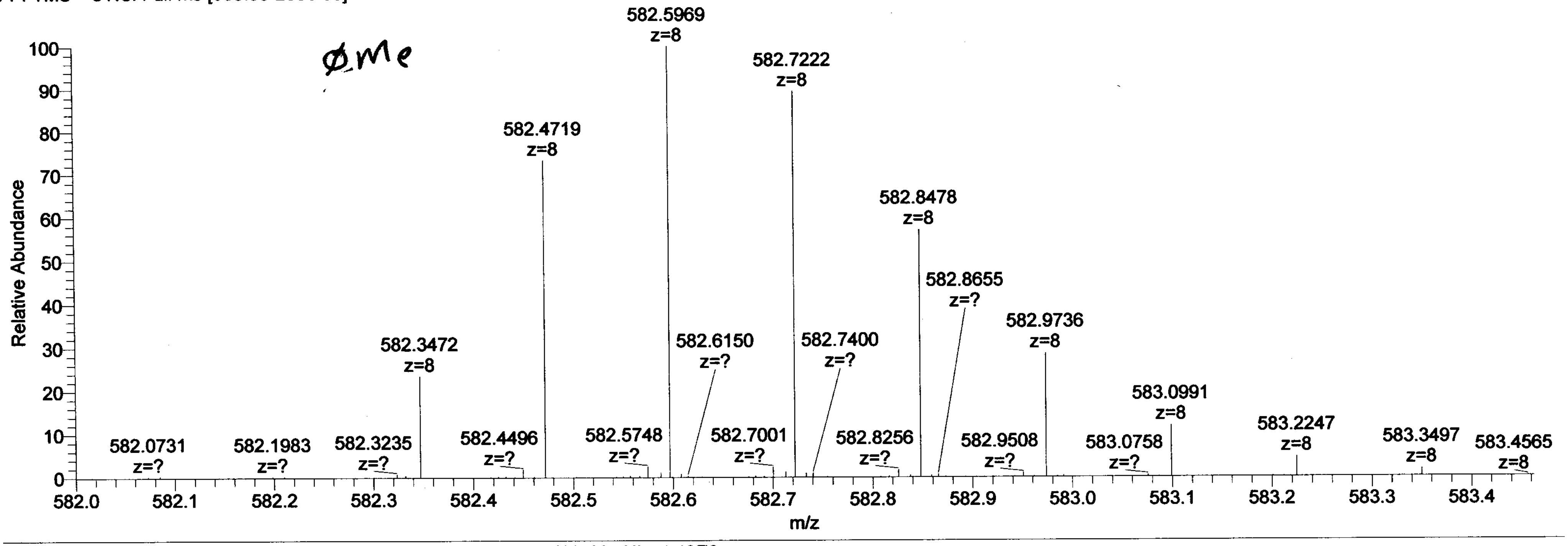

051010_jrc_TetmicH3_HILIC #20976-21157 RT: 169.71-170.87 AV: 38 NL: 4.40E6

F: FTMS + c NSI Full ms [300.00-2000.00]

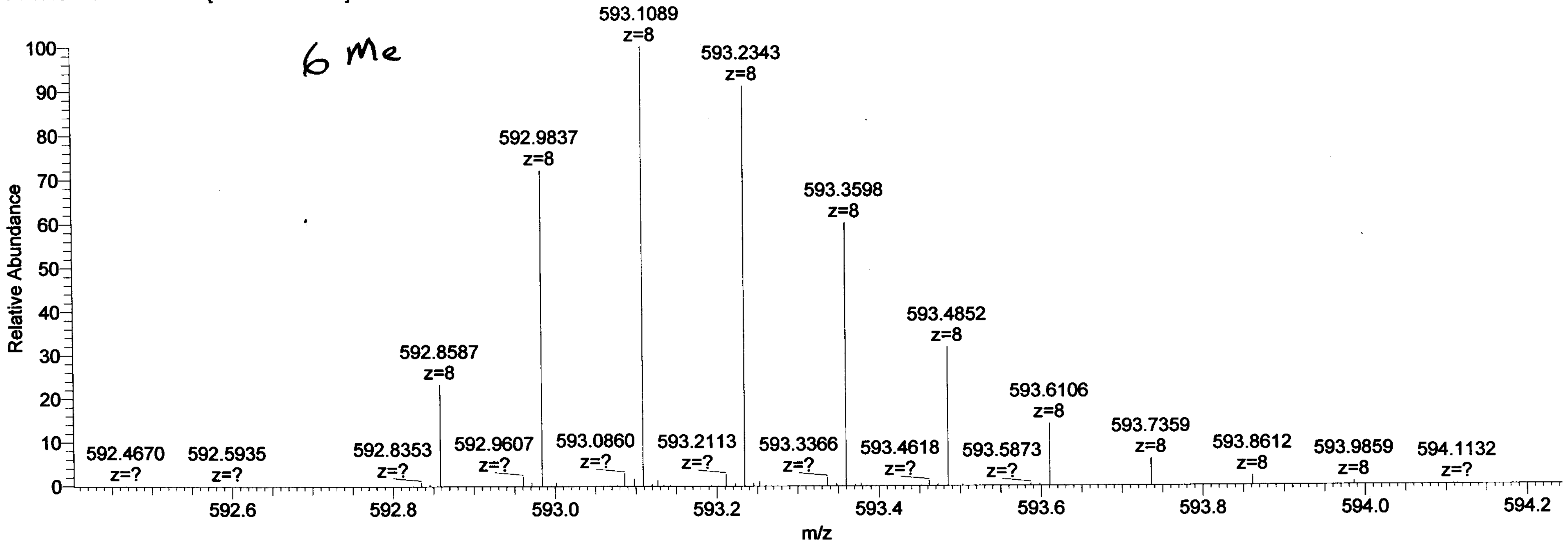

7 **ARKSTGAKAPRKQLASKAARKSAPATGGIKKPHRFRPGTVALRE** 50

9 14 18 23 27 36 37

Charge	Monoisotopic Mass	Average Mass
1	4651.7084	4654.49
2	2326.3578	2327.75
3	1551.2410	1552.17
4	1163.6826	1164.38
5	931.1475	931.70
6	776.1241	776.59
7	665.3932	665.79
8	582.3449	582.69
9	517.7518	518.06
10	466.0774	466.36
11	423.7983	424.05

Fragment Masses

+3 c ions	+2 c ions	+1 c ions		Sequence		+1 z ions	+2 z ions	+3 z ions
30.38	45.06	89.0709	1	A 7	44	4651.7084	2327.75	1552.17
82.44	123.16	245.1721 ✓	2	R	43	4564.6526	2284.20	1523.13
125.16	187.24	373.2670 ✓	3	K 9	42	4408.5515	2206.10	1471.07
154.19	230.78	460.2990 ✓	4	S	41	4280.4565	2142.02	1428.35
187.89	281.34	561.3467 ✓	5	T	40	4193.4245	2098.48	1399.32
206.91	309.86	618.3682 ✓	6	G	39	4092.3768	2047.92	1365.62 ✓
230.60	345.40	689.4053 ✓	7	A	38	4035.3553	2019.40	1346.60 ✓
273.33	409.49	817.5003 ✓	8	K 14	37	3964.3182	1983.86	1322.91 ✓
~~297.02~~	~~445.03~~	~~888.5374~~	~~9~~	A	36	3836.2233	1919.77 ✓	1280.18 ✓
329.39	493.59 ✓	985.5901 ✓	10	P	~~35~~	~~3765.1861~~	~~1884.23~~	~~1256.49~~
381.46	571.68 ✓	1141.6913 ✓	11	R	34	3668.1334	1835.67 ✓	1224.12 ✓
424.18	635.77 ✓	1269.7862 ✓	12	K 18	33	3512.0323	1757.58	1172.06 ✓
466.89	699.83 ✓	1397.8448 ✓	13	Q	32	3383.9373	1693.49 ✓	1129.33 ✓
504.61	756.41 ✓	1510.9289	14	L	31	3255.8787	1629.43 ✓	1086.62 ✓
528.30 ✓	791.95 ✓	1581.9660 ✓	15	A	30	3142.7947	1572.85	1048.90
557.33 ✓	835.49 ✓	1668.9980 ✓	16	S	29	3071.7575	1537.31 ✓	1025.21 ✓
600.05 ✓	899.58 ✓	1797.0930	17	K 23	28	2984.7255	1493.77 ✓	996.18 ✓
623.75 ✓	935.12	1868.1301	18	A	27	2856.6306	1429.68 ✓	953.46
647.44 ✓	970.66 ✓	1939.1672	19	A	26	2785.5934	1394.14 ✓	929.76
699.50 ✓	1048.75 ✓	2095.2683	20	R	25	2714.5563	1358.60 ✓	906.07 ✓
742.23 ✓	1112.84 ✓	2223.3633	21	K 27	24	2558.4552	1280.51 ✓	854.01 ✓
771.25 ✓	1156.38 ✓	2310.3953	22	S	23	2430.3603	1216.42 ✓	811.28
~~794.95~~	~~1191.92~~	~~2381.4324~~	~~23~~	A	22	2343.3282	1172.88 ✓	782.26 ✓
827.32 ✓	1240.48	2478.4852	24	P	~~21~~	~~2272.2911~~	~~1137.34~~	~~758.56~~
851.01	1276.02	2549.5223	25	A	20	2175.2383	1088.78 ✓	726.19
884.71	1326.57	2650.5700	26	T	19	2104.2012	1053.24 ✓	702.50 ✓
903.73	1355.09	2707.5914	27	G	18	2003.1536	1002.69 ✓	668.80 ✓
922.75	1383.62	2764.6129	28	G	17	1946.1321 ✓	974.16 ✓	649.78 ✓
960.47	1440.20	2877.6970	29	I	16	1889.1106 ✓	945.64 ✓	630.76 ✓
1003.19	1504.29	3005.7919	30	K 36	15	1776.0266 ✓	889.06 ✓	593.04 ✓
~~1045.92~~	~~1568.37~~	~~3133.8869~~	~~31~~	K 37	14	1647.9316 ✓	824.97 ✓	550.32 ✓
1078.29	1616.93	3230.9396	32	P	~~13~~	~~1519.8366~~	~~760.60~~	~~507.59~~
1124.01	1685.50	3367.9986	33	H	12	1422.7839 ✓	712.33 ✓	475.22
1176.07	1763.60	3524.0997	34	R	11	1285.7250 ✓	643.76 ✓	429.51
1225.13	1837.19	3671.1681	35	F	10	1129.6238 ✓	565.66 ✓	377.44
~~1277.19~~	~~1915.28~~	~~3827.2692~~	~~36~~	R	9	982.5554 ✓	492.07 ✓	328.38
1309.56	1963.84	3924.3220	37	P	~~8~~	~~826.4543~~	~~413.98~~	~~276.32~~
1328.58	1992.36	3981.3434	38	G	7	729.4016	365.42	243.95
1362.28	2042.92	4082.3911	39	T	6	672.3801 ✓	336.89	224.93
1395.33	2092.48	4181.4595	40	V	5	571.3324 ✓	286.34	191.23
1419.02	2128.02	4252.4966	41	A	4	472.2640 ✓	236.78	158.19
1456.74	2184.60	4365.5807	42	L	3	401.2269 ✓	201.24	134.49
1508.80	2262.70	4521.6818	43	R	2	288.1428 ✓	144.66	96.77
1552.17	2327.75	4651.7084	44	E 50	1	132.0417	66.56	44.71

051010_jrc_TetmicH3_HILIC 5/10/2010 12:20:05 PM

0 me

26 ul (~5pmole) Tet mic H3 GluC + 250 fmole AV and ACTH

051010_jrc_TetmicH3_HILIC #23419-24181 RT: 185.72-191.11 AV: 27 NL: 1.65E4

F: ITMS + c NSI d Full ms2 582.47@cid1.00 [150.00-2000.00]

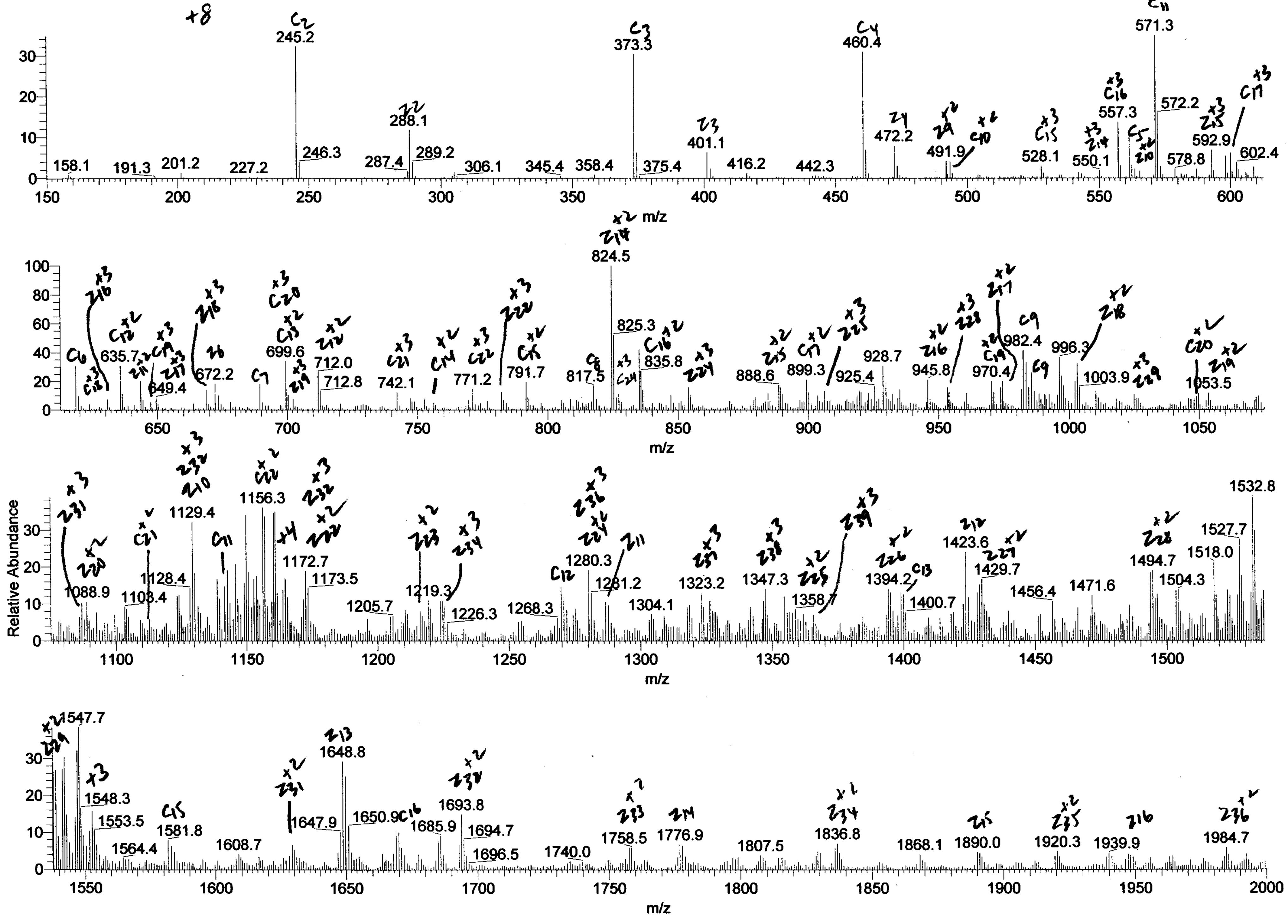

ARKSTGAKAPRKQLASKAARKSAPATGGIKKPHRFRPGTVALRE

Charge	Monoisotopic Mass	Average Mass
1	4735.8026	4738.65
2	2368.4049	2369.83
3	1579.2724	1580.22
4	1184.7061	1185.42
5	947.9663	948.54
6	790.1398	790.61
7	677.4066	677.81
8	592.8567	593.21
9	527.0956	527.41
10	474.4868	474.77
11	431.4432	431.70

Fragment Masses

+3 c ions	+2 c ions	+1 c ions		Sequence		+1 z ions	+2 z ions	+3 z ions
30.38	45.06	89.0709	1	A	44	4735.8026	2369.83	1580.22
82.44	123.16	245.1721	2	R	43	4648.7468	2326.28	1551.19
125.16	187.24	373.2670	3	K	42	4492.6457	2248.18	1499.12
154.19	230.78	460.2990	4	S	41	4364.5507	2184.10	1456.40
187.89	281.34	561.3467	5	T	40	4277.5187	2140.56	1427.37
206.91	309.86	618.3682	6	G	39	4176.4710	2090.00	1393.67
230.60	345.40	689.4053	7	A	38	4119.4495	2061.48	1374.65
273.33	409.49	817.5003	8	K	37	4048.4124	2025.94	1350.96
297.02	445.03	888.5374	9	A	36	3920.3175	1961.85	1308.24
329.39	493.59	985.5901	10	P	35	3849.2803	1926.31	1284.54
381.46	571.68	1141.6913	11	R	34	3752.2276	1877.75	1252.17
424.18	635.77	1269.7862	12	K	33	3596.1265	1799.66	1200.11
466.89	699.83	1397.8448	13	Q	32	3468.0315	1735.57	1157.38
504.61	756.41	1510.9289	14	L	31	3339.9729	1671.51	1114.67
528.30	791.95	1581.9660	15	A	30	3226.8889	1614.93	1076.95
557.33	835.49	1668.9980	16	S	29	3155.8517	1579.39	1053.26
614.08	920.62	1839.1401	17	K (+ 42)	28	3068.8197	1535.85	1024.23
637.77	956.16	1910.1772	18	A	27	2898.6777	1450.72	967.48
661.47	991.70	1981.2143	19	A	26	2827.6405	1415.18	943.79
713.53	1069.79	2137.3154	20	R	25	2756.6034	1379.64	920.10
770.28	1154.92	2307.4575	21	K (+ 42)	24	2600.5023	1301.55	868.03
799.31	1198.46	2394.4895	22	S	23	2430.3603	1216.42	811.28
823.00	1234.00	2465.5266	23	A	22	2343.3282	1172.88	782.26
855.37	1282.56	2562.5794	24	P	21	2272.2911	1137.34	758.56
879.07	1318.10	2633.6165	25	A	20	2175.2383	1088.78	726.19
912.77	1368.65	2734.6642	26	T	19	2104.2012	1053.24	702.50
931.79	1397.17	2791.6856	27	G	18	2003.1536	1002.69	668.80
950.80	1425.70	2848.7071	28	G	17	1946.1321	974.16	649.78
988.52	1482.28	2961.7912	29	I	16	1889.1106	945.64	630.76
1031.25	1546.37	3089.8861	30	K	15	1776.0266	889.06	593.04
1073.97	1610.46	3217.9811	31	K	14	1647.9316	824.97	550.32
1106.35	1659.01	3315.0338	32	P	13	1519.8366	760.88	507.59
1152.06	1727.58	3452.0928	33	H	12	1422.7839	712.33	475.22
1204.12	1805.68	3608.1939	34	R	11	1285.7250	643.76	429.51
1253.18	1879.27	3755.2623	35	F	10	1129.6238	565.66	377.44
1305.24	1957.36	3911.3634	36	R	9	982.5554	492.07	328.38
1337.62	2005.92	4008.4162	37	P	8	826.4543	413.98	276.32
1356.63	2034.45	4065.4376	38	G	7	729.4016	365.42	243.95
1390.33	2085.00	4166.4853	39	T	6	672.3801	336.89	224.93
1423.38	2134.56	4265.5537	40	V	5	571.3324	286.34	191.23
1447.07	2170.10	4336.5908	41	A	4	472.2640	236.78	158.19
1484.79	2226.68	4449.6749	42	L	3	401.2269	201.24	134.49
1536.85	2304.78	4605.7760	43	R	2	288.1428	144.66	96.77
1580.22	2369.83	4735.8026	44	E	1	132.0417	66.56	44.71

051010_jrc_TetmicH3_HILIC 5/10/2010 12:20:05 PM

26 ul (~5pmole) Tet mic H3 GluC + 250 fmole AV and ACTH

051010_jrc_TetmicH3_HILIC #20976-21148 RT: 169.77-170.47 AV: 6 NL: 2.30E4

F: ITMS + c NSI d Full ms2 592.98@cid1.00 [150.00-2000.00]

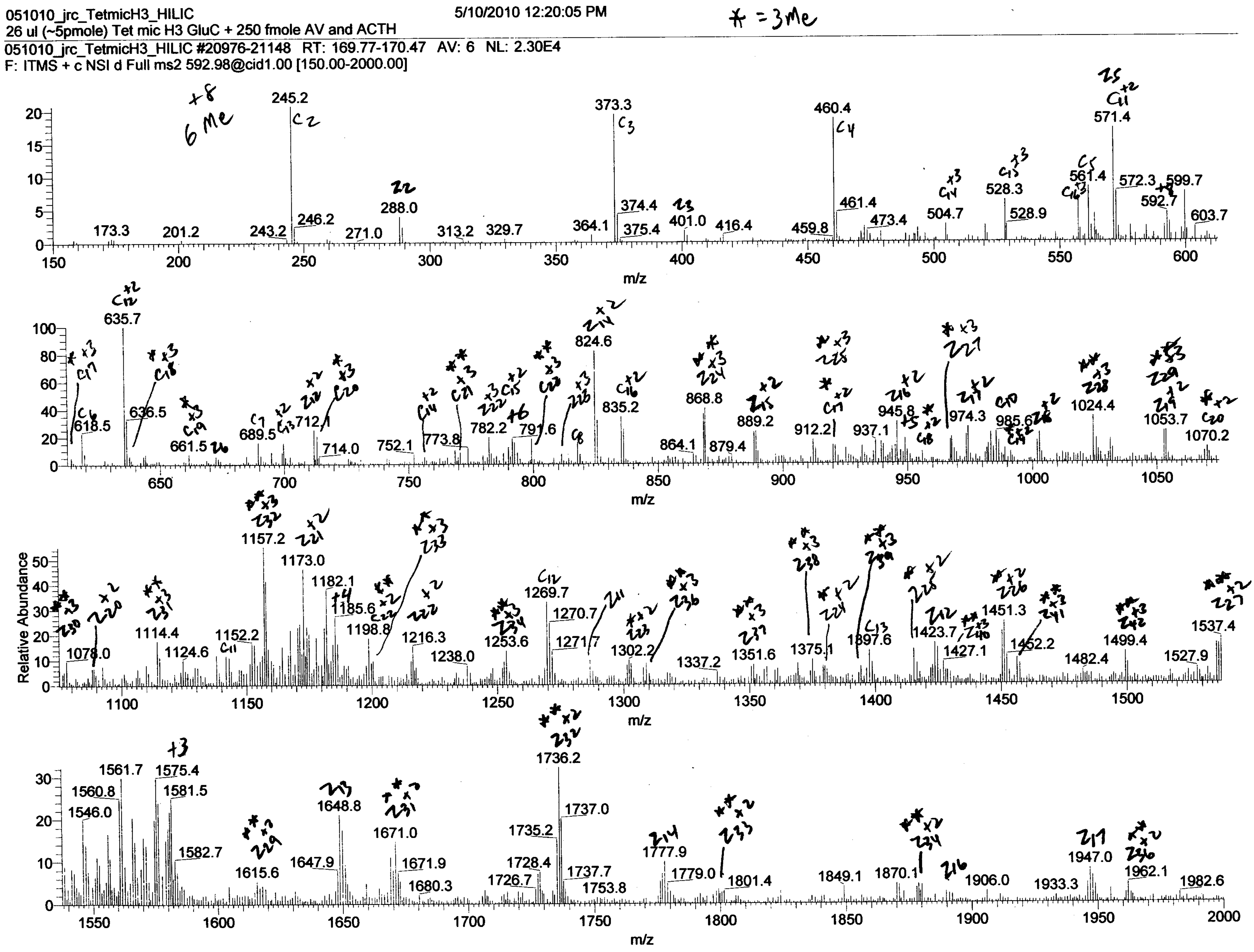

Supplement: Figure 1—source data 1. — DOI: http://dx.doi.org/10.7554/eLife.02996.004 [file elife02996s001.pdf]
